# Serum Amyloid A3 Promoter-Driven Luciferase Activity Enables Visualization of Diabetic Kidney Disease

**DOI:** 10.3390/ijms23020899

**Published:** 2022-01-14

**Authors:** Tolulope Peter Saliu, Nao Yazawa, Kotaro Hashimoto, Kenshu Miyata, Ayane Kudo, Mayu Horii, Mion Kamesawa, Thanutchaporn Kumrungsee, Noriyuki Yanaka

**Affiliations:** Graduate School of Integrated Sciences for Life, Hiroshima University, 1-4-4 Kagamiyama, Higashi, Hiroshima 739-8528, Japan; d195213@hiroshima-u.ac.jp (T.P.S.); m206855@hiroshima-u.ac.jp (N.Y.); 191395.kon.rec@gmail.com (K.H.); b184262@hiroshima-u.ac.jp (K.M.); b196517@hiroshima-u.ac.jp (A.K.); b192799@hiroshima-u.ac.jp (M.H.); b182885@hiroshima-u.ac.jp (M.K.); kumrung@hiroshima-u.ac.jp (T.K.)

**Keywords:** diabetic nephropathy, streptozotocin, STZ-induced diabetic model, in vivo bioluminescence, in vivo imaging, serum amyloid A3

## Abstract

The early detection of diabetic nephropathy (DN) in mice is necessary for the development of drugs and functional foods. The purpose of this study was to identify genes that are significantly upregulated in the early stage of DN progression and develop a novel model to non-invasively monitor disease progression within living animals using in vivo imaging technology. Streptozotocin (STZ) treatment has been widely used as a DN model; however, it also exhibits direct cytotoxicity to the kidneys. As it is important to distinguish between DN-related and STZ-induced nephropathy, in this study, we compared renal responses induced by the diabetic milieu with two types of STZ models: multiple low-dose STZ injections with a high-fat diet and two moderate-dose STZ injections to induce DN. We found 221 genes whose expression was significantly altered during DN development in both models and identified serum amyloid A3 (*Saa3*) as a candidate gene. Next, we applied the Saa3 promoter-driven luciferase reporter (Saa3-promoter luc mice) to these two STZ models and performed in vivo bioluminescent imaging to monitor the progression of renal pathology. In this study, to further exclude the possibility that the in vivo bioluminescence signal is related to renal cytotoxicity by STZ treatment, we injected insulin into Saa3-promoter luc mice and showed that insulin treatment could downregulate renal inflammatory responses with a decreased signal intensity of in vivo bioluminescence imaging. These results strongly suggest that Saa3 promoter activity is a potent non-invasive indicator that can be used to monitor DN progression and explore therapeutic agents and functional foods.

## 1. Introduction

Diabetic nephropathy (DN), also referred to as diabetic kidney disease, is the progressive development of renal insufficiency induced by the diabetic milieu [1]. Approximately 40% of patients diagnosed with either type 1 or type 2 diabetes develop DN, and this number is projected to increase dramatically with the increasing trend of diabetes worldwide [2,3]. Currently, DN research relies on traditional diagnostic techniques, such as the histological assessment of collagen deposition and quantification of serum/urine biomarkers (e.g., creatinine, blood urea nitrogen (BUN), albumin, and cytokines) to confirm kidney disorders [3]. Although these markers have provided essential insight into DN progression in human patients, there are problems of low sensitivity and missing early injury responses in diabetic kidneys. In addition, these traditional serum/urine biomarkers are often affected by other factors such as age, muscle mass, protein diet, and intake of certain drugs [4,5]. Thus, new biomarkers that are suitable for capturing the characteristics of early kidney injury and can easily obtain reliable data are being searched and are expected to provide essential insight into DN progression. Accumulating evidence has shown that chronic renal inflammation and fibrosis are hallmark pathological features of DN [1,6,7,8,9]. Thus, the assessment of renal inflammation and fibrosis using non-invasive tests will become more important in diagnosing DN at an earlier stage and efficiently analyzing new therapeutic agents and functional foods. In vivo imaging is a promising technology for the non-invasive monitoring of disease progression within living animals and/or for examining the efficacy of emerging therapeutic strategies with the same individual mice in real-time [10,11]. Non-invasive and high-resolution bioluminescence imaging tools for studying biological processes and quantitatively monitoring disease progression are now being developed and have attracted attention in various research fields [10]. In our previous study, we successfully applied the serum amyloid A3 (Saa3) gene promoter-luciferase (luc) reporter to non-invasively monitor low-grade inflammation in the white fat tissues of obese mice fed with a high-fat diet and in a dietary adenine-induced tubulointerstitial injury model [12,13]. Here, since we found that Saa3 expression is significantly upregulated in diabetic kidneys in two different streptozotocin-induced DN models without direct cytotoxicity to the kidneys, we used a novel in vivo Saa3-promoter bioluminescence imaging technique to monitor renal pathology in these two DN models. We further performed mRNA expression analyses to confirm whether the bioluminescence signal reflected the degree of kidney damage from hyperglycemia. Our present study suggests that novel in vivo bioluminescence imaging could be useful in the non-invasive visualization of pathophysiological changes in the kidney that characterize DN in real-time.

## 2. Results

### 2.1. Gene Expression Patterns of Saa3 and Other Fibro-Inflammatory Markers Are Altered with DN Development

**Figure 1:**
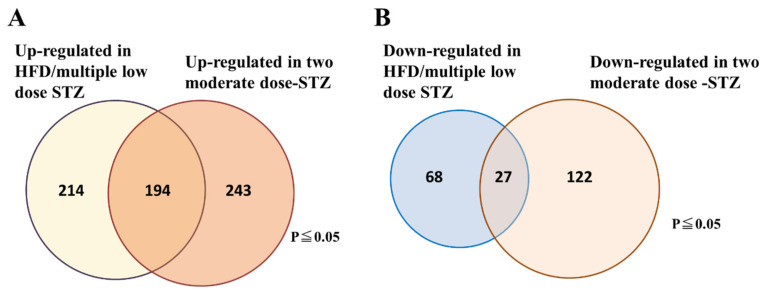
Venn diagram showing the number of genes that are significantly altered in the kidney tissues of the two STZ-induced DN models. (**A**) Number of upregulated genes. (**B**) Number of down-regulated genes. The data are representative of two independent experiments (i.e., HFD/multiple low-dose STZ-induced DN model, and two-moderate-dose STZ-induced DN model).

To examine the molecular signature that can characterize an early stage of DN in streptozotocin (STZ)-induced DN models, we performed DNA microarray analyses of kidneys from two STZ-induced DN models using C57BL/6 mice. In the first model, DN was induced by a combination of a high-fat diet and multiple low-dose STZ injections, which is a well-established DN model without renal cytotoxicity induced by STZ treatment. In general, high doses of STZ have a non-specific cytotoxic effect that has been shown to cause acute kidney damage in mice and rats [14,15]. A high-dose STZ injection often makes it difficult to interpret any observations of nephropathy because hyperglycemia-induced renal injuries are confused with acute renal cytotoxicity caused by STZ treatment. To solve this problem, previous studies have developed two moderate-dose STZ injections (2 × 125 mg/kg per day STZ) to establish diabetes in C57BL/6 mice. These injections led to a mild resistance to STZ, suggesting that renal damage in this STZ model is not related to the acute tubular cytotoxicity seen in those undergoing continuous insulin administration to prevent renal pathology [16]. Therefore, we adopted this STZ-induced DN model as the second model in this study. In these two STZ models, we found that the expression of 221 genes was similarly altered, with 194 genes upregulated and 27 genes downregulated in both DN models (Figure 1A,B). Functional classification of the genes indicated that numerous genes were associated with inflammation and fibrosis, whereas other genes with robust induction were associated with senescence and apoptosis (Table 1).

**Figure 2:**
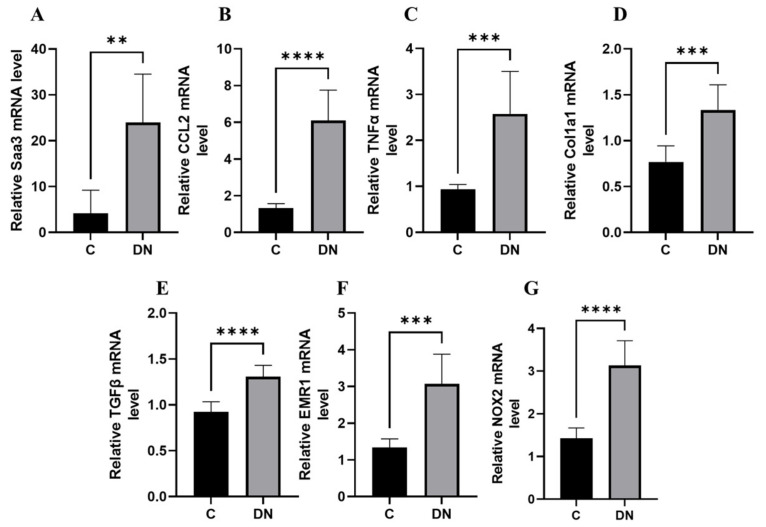
Renal fibro-inflammatory markers are upregulated in HFD/multiple low-dose STZ-induced DN model (**A**–**G**) Total RNAs in kidney tissues from the HFD/multiple low-dose STZ-induced DN model (*n* = 9) were isolated. The relative mRNA expression level of each gene was determined by quantitative PCR and normalized to L19 mRNA level and are presented as means ± S.E. ** *p* < 0.01, *** *p* < 0.001, **** *p* < 0.0001. The data are representative of two independent experiments. DN = Diabetic Nephropathy, C = Control.

We performed a transcriptomic analysis of fibro-inflammatory marker genes Tumour Necrosis Factor alpha (*TNFα*), Transforming growth factor beta (*TGFβ*), Collagen, type I, alpha1 (*Col1a1*), chemokine (C-C motif) ligand 2 (*Ccl2*), serum amyloid A3 (*SAA3*), EGF-like module-containing mucin-like hormone receptor-like 1 (*EMR1*), and NADPH oxidase 2 (*NOX2*) using qPCR on renal tissues from the HFD/multiple low-dose STZ model. We found that the expression of these fibro-inflammatory markers was highly upregulated in the HFD/multiple low-dose STZ model. (Figure 2A–G). In particular, Saa*3* mRNA expression was significantly upregulated in the renal tissue of the HFD/multiple low-dose STZ-induced DN model (5-fold increase), as validated through qPCR (Figure 2A). Interestingly, previous studies have shown that Saa3 plays an active role in inflammatory disorders, and an increase in its expression is accompanied by a concomitant increase in inflammatory biomarker genes in the renal tissue of patients with DKD and corresponding diabetic mouse models [17,18,19]. These findings strongly suggest that Saa3 promoter activity is a useful biomarker for monitoring renal pathology in DN.

### 2.2. Non-Invasive High-Resolution Bioluminescence Imaging Detected Diabetes Kidney Disease in the HFD/Multiple Low-Dose STZ-Induced DN Model

**Figure 3:**
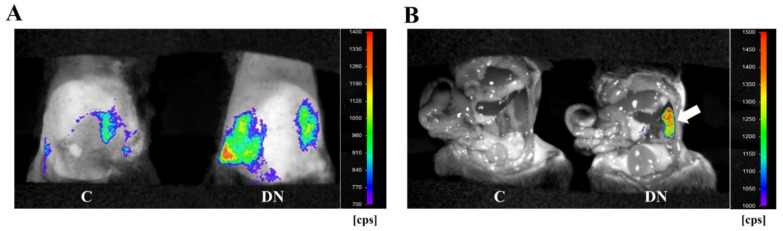
Visualization of renal pathology in HFD/multiple low-dose STZ-induced DN using Saa3 promoter-luc mice. (**A**) In vivo bioluminescence imaging from the back of Saa3 promoter-luc mice shows a strong intensity of bioluminescent signal (from violet for least intense to red for most intense), reflecting kidney injury. (**B**) Bioluminescent analysis of mouse organs exposed to bioluminescent imaging confirmed that the intense bioluminescent signal generated was specifically from the diabetic induced injured kidney (the white arrow), and not from the adjacent organs of the Saa3 promoter-luc mice that were induced with HFD/multiple low-dose STZ. DN = Diabetic Nephropathy, C = Control.

To monitor diabetic kidney disease in the HFD/multiple low-dose STZ-induced DN model, we performed in vivo bioluminescence imaging with Saa3 promoter-luciferase transgenic mice (Saa3 promoter-luc mice) eight weeks after diabetic induction. As shown in Figure 3A, the bioluminescent signal from the renal tissues in HFD/multiple low-dose STZ mice was stronger (from violet for the least intense to red for the most intense) when compared to the normal kidney from control mice. To verify if the visualized bioluminescence signal was specifically generated from the injured kidney in HFD/multiple low-dose STZ mice, we performed in vivo bioluminescence analysis after opening the stomachs of Saa3 promoter-luc mice. Our results showed that the Saa3-mediated bioluminescent signal was specifically detected in the injured kidney (white arrow), and not in the adjacent organs in the HFD/multiple low-dose STZ-induced DN model, whereas the uninjured kidneys and other organs of the control mice showed no bioluminescent signal (Figure 3B).

### 2.3. Histological, Biochemical, and Molecular Validation of In Vivo Bioluminescence Signals from the Renal Tissues of Two-Moderate-Dose STZ-Induced DN Model

**Figure 4:**
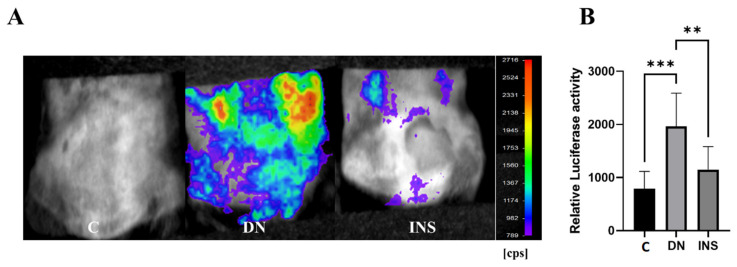
Bioluminescence imaging reveals renal pathology and therapeutic response of insulin in two-moderate-dose STZ-induced DN model using Saa3 promoter-luc mice. (**A**) The in vivo bioluminescence imaging from the back of Saa3 promoter-luc mice shows intense bioluminescence signal from the kidney tissues and how insulin treatment decreased the signal intensity, reflecting less severe injury in kidney of the insulin-treated mouse induced with two moderate-dose STZ injections (*n* = 5). (**B**) Quantitative data (*n* = 6). Data are presented as means ± S.E. ** *p* < 0.01, *** *p* < 0.001 as determined by ANOVA followed by Dunnett’s test. DN = Diabetic Nephropathy, C = Control, INS = Insulin treatment.

To investigate whether the Saa3-mediated bioluminescence signal was also able to detect the DN status of STZ-induced DN model at a moderate dose (2 × 125 mg/kg), Saa3 promoter-luc mice were subjected to *C* bioluminescence imaging after four weeks of diabetic induction. As shown in Figure 4A, the bioluminescence signal from diabetes-induced kidney injury in moderate-dose STZ-induced mice was extremely strong; meanwhile, there was no bioluminescent signal in the control group, reflecting an uninjured kidney. Quantitative analysis of bioluminescence intensity from two moderate-dose STZ-induced mouse kidneys showed a 2.5-fold increase in luciferase activity compared to the control (Figure 4B).

Interestingly, the bioluminescence signal was significantly reduced in moderate-dose STZ-induced mice after insulin treatment (Figure 4A,B), thereby suggesting the reliability of using the Saa3 promoter-luc mouse kidney in monitoring DN progression and exploring therapeutic agents and functional foods.

**Figure 5:**
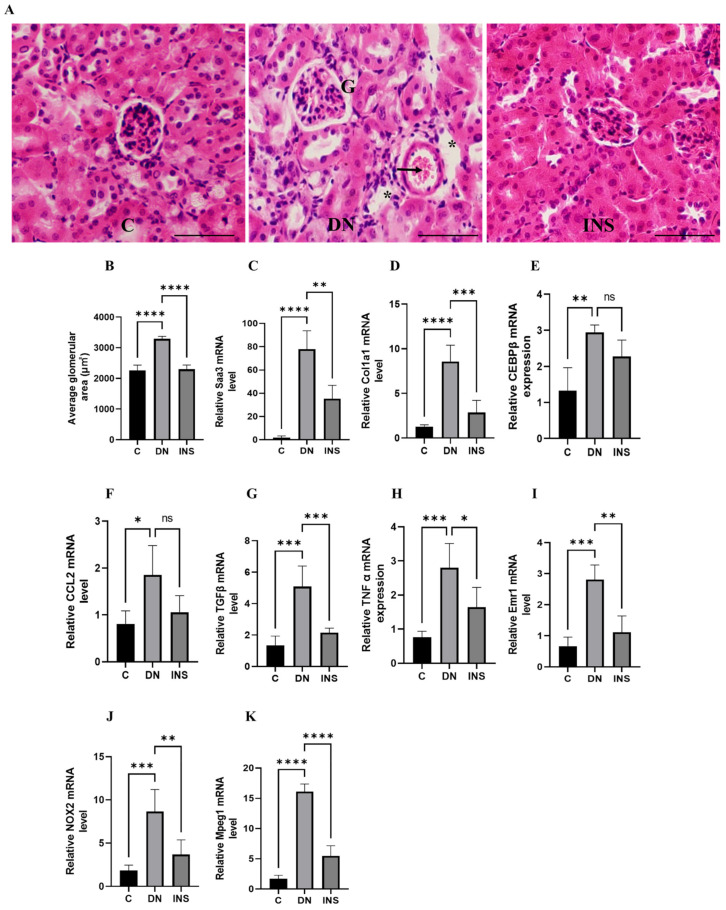
Validation of bioluminescence signals of Saa3 promoter-luc mice with traditional diagnostic techniques and ameliorative effect of insulin therapy on renal fibro-inflammatory cues in two-moderate-dose STZ-induced DN model. (**A**) Representative image of the kidney histological staining with H&E reagents. Glomerular hypertrophy, glomerular hypercellularity (G), brush border disruption (asterisks), and interstitial hemorrhage (arrow) were significantly increased in renal tissue of DN mice. Scale bar = 200 µm. (**B**) Quantification of average glomerular size (*n* = 5). (**C**–**K**) Kidney gene expression analysis of fibro-inflammatory markers in two-moderate-dose STZ-induced DN model (*n* = 5). The relative mRNA expression level of each gene was determined by quantitative PCR and normalized to L19 mRNA level and are presented as means ± S.E. * *p* < 0.05, ** *p* < 0.01, *** *p* < 0.001, **** *p* < 0.0001 as determined by ANOVA followed by Dunnett’s test; ns: statistically not significant. DN = Diabetic Nephropathy, C = Control, INS = Insulin treatment.

Moreover, to validate the in vivo bioluminescent results, we screened the renal tissues of the same Saa3 promoter-luc mice induced with moderate-dose STZ and their corresponding control groups, which were subjected to in vivo bioluminescence imaging. Histological assessment of diabetes-induced injured renal tissues revealed that glomerular hypertrophy, glomerular hypercellularity, brush border disruption, and interstitial hemorrhage were significantly increased. Notably, the histological renal injury parameters in moderate-dose STZ mice were ameliorated by insulin treatment (Figure 5A,B). Furthermore, since our microarray data (Table 1) have implicated inflammation and fibrosis as common cardinal pathogenetic mechanisms that promote diabetic nephropathy, we performed transcriptomic analysis of the fibro-inflammatory marker genes (TNFα, TGFβ, *Col1a1, Ccl2, Saa3,* CCAAT/ enhancer binding protein β (*C/EBP β*)*, EMR1,* Macrophage-expressed gene 1 (*Mpeg1*)*,* and *NOX2*) in the tissues of the same kidney. The results showed that the mRNA expression levels of *TNFα, TGFβ, Col1a1*, *Ccl2, Saa3, C/EBP β, EMR1, NOX2,* and *Mpeg1* were significantly upregulated (Figure 5C–K). Notably, the mRNA expression levels of *TNFα, CCL2, Emr1, TGFβ,* and *Col1a1* were positively correlated with in vivo luciferase activity (*r* = 0.927, *p* < 0.05; *r* = 0.821, *p* = 0.08; *r* = 0.978, *p* < 0.01; *r* = 0.897, *p* < 0.05; *r* = 0.819, *p* = 0.08, respectively; Supplementary Figure S1A–E, see Supplementary Materials). These results further suggested that Saa3-luciferase mice can be applied as a non-invasive model to monitor fibro-inflammation the key molecular driver of diabetic nephropathy.

Interestingly, despite the robust induction of fibro-inflammatory markers in the moderate-dose STZ model, insulin treatment significantly reduced the expression of kidney fibro-inflammatory markers (Figure 5C–K).

**Figure 6:**
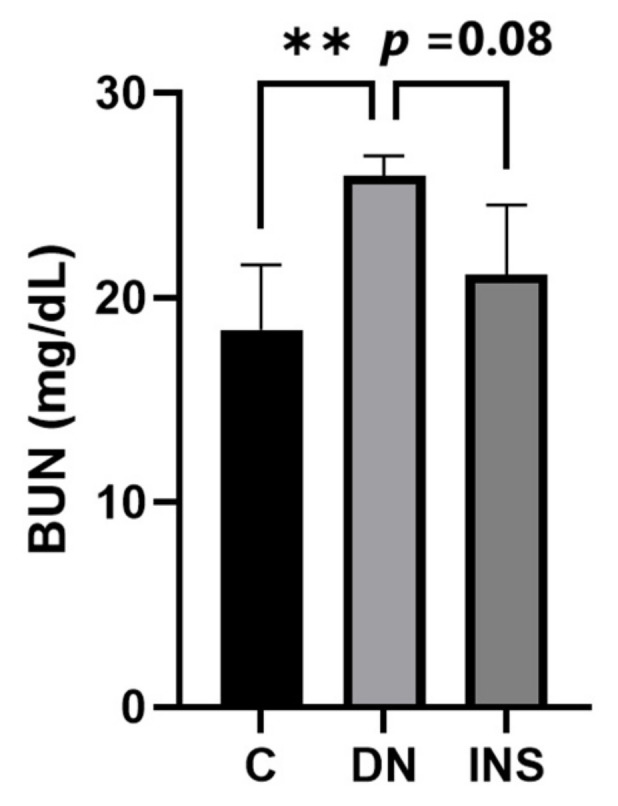
Elevated plasma BUN level in two-moderate-dose STZ-induced DN model was suppressed by insulin treatment (*n* = 5). Data are presented as means ± S.E. ** *p* < 0.01 as determined by ANOVA followed by Dunnett’s test. DN = Diabetic Nephropathy, C = Control, INS = Insulin treatment.

We also reported a biochemical analysis of the plasma BUN concentration. We found that the plasma BUN concentration significantly increased in moderate-dose STZ-induced DN Saa3 promoter-luc mice as compared to their corresponding control group. However, insulin treatment decreased the plasma BUN concentration (Figure 6). Taken together with our histological and molecular findings, these results indicate that diabetes-induced kidney injury mediated by fibro-inflammatory cues can be successfully monitored using non-invasive Saa3-promoter bioluminescence imaging.

## 3. Discussion

Diabetic nephropathy (DN) is a common microvascular complication of diabetes and a major cause of end-stage renal disease worldwide [2,20]. Traditional techniques, such as the histological assessment of collagen deposition and quantification of serum/urine biomarkers (e.g., creatinine, blood urea nitrogen (BUN), albumin, and cytokines), are the most commonly used to diagnose kidney disorders [3]. Although these markers have provided essential insight regarding renal functions in human patients and animals, signals of early responses of renal pathologies are often missed by these traditional methods of monitoring renal functions. Serum creatinine and BUN are easy to measure using specific assays; however, these markers are often influenced by various factors, such as diet and other tissue functions [4,5]. Despite their shortcomings, the demand for early diagnostic markers is expected to increase in human patients and animal experiments. In this study, we attempted to identify the molecular signature of the renal inflammatory responses induced by the diabetic milieu using two types of STZ-induced DN models. STZ is widely used as a diabetogenic agent in rodent models of DN [15,21,22]. Since STZ is an analog of glucose, it is efficiently taken up by pancreatic beta cells via the glucose transporter Glut2 and causes DNA damage and beta-cell death [15,21,22]. However, because STZ treatment is toxic to other tissues, such as the kidney, numerous studies have attempted to clarify its undesirable side effects on other tissues, particularly by high-dose treatment with STZ [15,21,22,23]. In this study, to eliminate the detrimental toxicity to the kidney, we used two types of STZ models, HFD/multiple low-dose STZ injections and two moderate-dose STZ injections that can promote DN development by inducing hyperglycemia. We then performed the comparative transcriptome analyses of damaged kidneys from both STZ models. Consistent with previous studies [18,24,25,26,27,28], these data indicate significant increases in fibrosis and inflammatory marker mRNA levels in both STZ-induced DN models. In this study, we finally utilized the Saa3-promoter activity as a sensitive and specific tool for detecting and visualizing renal pathology induced by diabetic fibro-inflammatory cues in real-time via non-invasive in vivo bioluminescence imaging. This study suggests that this novel bioluminescence imaging tool can be used not only to detect DN status in the two well-established STZ-induced models of DN but also to monitor therapeutic responses in the same individual mice.

Fibro-inflammatory cues are the cardinal molecular signals that drive DN pathogenesis and progression [1,6,7,8,9,29,30]. Notably, several studies have shown that serum amyloid A (Saa) is a key potential mediator of danger signals that influence inflammatory processes in several chronic inflammatory diseases, such as rheumatoid arthritis, atherosclerosis, obesity, and kidney diseases [17,31,32,33,34]. The Saa3 protein, one of the subtypes of the Saa family, was originally characterized as an acute-phase protein [35]. For example, Saa3 is highly expressed in adipose tissue in obese mice, which thus possibly plays a role in monocyte chemotaxis, providing a mechanism for macrophage accumulation that occurs during obesity development [34]. In addition, Saa can mediate a cascade of inflammatory events via interactions with multiple receptors, including Toll-like receptor 2 (TLR2), TLR4, scavenger receptor class B type 1 (SR-B1), and receptor for advanced glycation end-product (RAGE) [17]. However, the pathological relationship between Saa3 and diabetes-induced renal fibro-inflammation has not been fully elucidated. Various inflammatory stimuli, such as TNF-α, CCL-2, IL-1β, and IL-6, which drive chronic renal inflammation in diabetic kidney disease, have been shown to stimulate Saa3 production in mice [36]. Interestingly, a recent report suggested that the accumulation of advanced glycation end-products (AGEs) in a hyperglycemic environment can increase Saa3 mRNA expression in podocytes, which are located around the capillaries of the glomerulus and further contribute to the production of pro-inflammatory cytokines through increased Saa3 expression [18,37,38]. These results suggested a mechanistic pathway for the induction of renal inflammation under diabetic conditions in mice. Notably, the elevated levels of the Saa family seen in the two different STZ models are similar to those observed in the kidneys of human patients with DN [18,19]. Taken together, these findings suggest that Saa3 plays an important role in inflammatory and fibrotic disorders in DN, and monitoring Saa3 promoter activity in renal tissues may be useful for the evaluation of diabetic kidney disease.

As described above, inflammation and fibrosis are essential factors that can promote both the pathogenesis and progression of DN. Despite the growing trend of this life-threatening disease, non-invasive screening, and monitoring tools for diabetes-induced fibro-inflammation in renal tissues in vivo are limited. Further experiments are needed to determine whether the in vivo bioluminescence signal in these two STZ-induced DN models is directly associated with increased fibro-inflammatory signals from diabetes-induced renal injury and to identify factors related to increased renal Saa3 mRNA expression during DN development. These factors are more important in driving Saa3 promoter activity in Saa3-luc mice by analyzing the causal relationship with glomerular hypertrophy observed using fibro-inflammatory markers (TNFα, TGFβ, Col1a1, Ccl2, EMR1, Nox2, and Mpeg1), which are known to cause progressive renal injuries in both animal models and human patients. Owing to the low sensitivity of these traditional serum/urine biomarkers, they tend to miss early injury responses in the diabetic kidneys of mice. This novel in vivo bioluminescence imaging is expected to serve as an important pre-clinical tool with animal studies, and will be useful for the non-invasive, real-time monitoring of the effectiveness of drugs or food factors for DN progression.

## 4. Materials and Methods

### 4.1. Experimental Animals

Seven-week-old male C57BL/6J mice (Charles River Japan, Hino, Japan) and Saa3-promoter luc mice were used in this study. All mice were housed in a temperature-controlled (24 ± 1 °C) room with a 12 h/12 h light/dark cycle. The mice had free access to food and water. All animal experiments were approved by the Hiroshima University Animal Care and Use Committee (Permit Number: C18-15-5).

### 4.2. Diabetic Nephropathy Animal Models

Saa3-promoter transgenic mice (Saa3-promoter luc mice) carrying mouse Saa3 promoter regions (−314/+50) upstream of full-length luciferase cDNA were obtained and maintained as previously described [12,13]. Both the male C57BL/6J (WT) mice and Saa3-promoter luc mice were used to develop two streptozotocin (STZ)-induced DN models. For the first STZ-induced DN model, all mice were divided into two groups: the control group and the DN group. The control group was fed a standard laboratory chow diet, and the DN group was fed a high-fat diet (HFD) (Oriental Yeast Co., Tokyo, Japan; 45% fat, 20.5% protein, and 34.8% carbohydrate) together with low-dose STZ (55 mg/kg for 5 consecutive days) (HFD/multiple-dose STZ-induced DN group). Notably, the HFD/multiple-dose STZ-induced DN mouse group was initially fed HFD for 4 weeks before being injected with low-dose STZ. Animals in the first model were analyzed 12 weeks after low-dose STZ treatment. For the second model of STZ-induced DN, all mice were divided into three groups: the control group, the DN group, and the insulin-treated DN group. Diabetes was induced via intraperitoneal (i.p.) injections of streptozotocin (STZ, Sigma-Aldrich, St Louis, MO, USA) at 125 mg/kg/day for 2 consecutive days (two moderate-dose STZ-induced DN group). Mice in the control group were administered citrate buffer alone. All mice were maintained on a standard laboratory chow diet (Oriental Yeast, Tokyo, Japan) and analyzed four weeks after two moderate-dose STZ treatments. In this model, only the mice with fasting blood glucose levels of ≥300 mg dL^−1^ after STZ injection were used for further studies.

### 4.3. DNA Microarray

Microarray analyses were performed as previously described [12]. Briefly, total RNA was extracted from the renal tissues of WT mice in both models of STZ-induced DN using the RNeasy lipid tissue kit (Qiagen Sciences, Germantown, MD, USA). The RNA samples were subjected to cRNA synthesis for DNA microarray analysis (44 K whole mouse genome 60-mer oligo microarray, Agilent Technologies, Palo Alto, CA, USA). Fluorescence labeling, hybridization, and image processing were performed according to the manufacturer’s instructions. Statistical analysis of the gene expression data was performed using the Agilent Feature Extraction software (version 9.5) (Santa Clara, CA, USA).

### 4.4. RT-PCR

One microgram of total RNA isolated from the renal tissues of WT mice induced by HFD/multiple low-dose STZ and Saa3-promoter luc mice induced with two moderate doses of STZ was reverse transcribed into cDNA using ReverTra Ace (TOYOBO, Osaka, Japan) and random hexamers (TaKaRa Bio, Kyoto, Japan), according to the manufacturer’s instructions. Quantitative PCR reactions were then performed on StepOnePlusTM (Applied Biosystems, Foster City, CA, USA) using THUNDERBIRD SYBR qPCR Mix reagents (TOYOBO) under previously described conditions [13]. The primers used for the PCR analysis are listed in Table 2. The expression level of the target gene was normalized to that of the housekeeping gene L19. The relative gene expression was calculated using the comparative Ct (2^−ΔΔCt^) method.

### 4.5. In Vivo Bioluminescent Imaging

In vivo bioluminescence imaging was performed as previously described [12,13]. Briefly, before acquiring images, 150 mg/kg body weight D-luciferin (Promega, Madison, WI, USA) was administered intraperitoneally to all Saa3 promoter-luc mice in both models five minutes after substrate administration using the NightOWL II Imaging Systems LB983 (Berthold Technologies, Bad Wildbad, Germany). For each mouse, the signal intensity was quantified as the sum of all detected photon counts per second and was presented as counts/s (cps). All images were adjusted to the same scale as the minimum and maximum luminescent intensities for any given analysis.

### 4.6. Histological Analysis

Renal tissues from Saa3-promoter luc mice induced with two moderate doses of STZ were fixed in 4% formalin solution and embedded in paraffin, and 5 mm-thick sections were prepared. The sections were then processed for hematoxylin and eosin (H&E) staining to determine the overall histology according to standard procedures [39].

### 4.7. Measurement of Plasma BUN Level

The Plasma BUN levels of Saa3-promoter luc mice induced with two moderate doses of STZ were determined using a Beckman Coulter AU480 analyzer (Beckman Coulter, Krefeld, Germany), which is an automated chemistry instrument for turbidimetric, spectrophotometric, and ion-selective electrode measurements.

### 4.8. Statistical Analysis

Data are presented as means ± S.E. Differences in significance levels between the compared groups were determined by a Student’s *t*-test or one-way ANOVA followed by Dunnett’s test. Results were considered statistically significant at *p* < 0.05 (denoted appropriately in all figures).

## Figures and Tables

**Table 1:** Representative upregulated genes in both models (*p* ≤ 0.05).

Gene Symbol	Gene Description	Fold in HFD/Multiple Low-Dose STZ-Induced DN	Fold in Two Moderate-Dose STZ-Induced DN
Inflammation and immune response			
*Ccl7*	chemokine (C-C motif) ligand 7	9.57	9.21
*C3*	complement component 3	8.32	4.03
*Cxcl1*	chemokine (C-X-C motif) ligand 1	6.02	2.96
*Cxcl13*	chemokine (C-X-C motif) ligand 13	5.07	3.72
*Ccl2*	chemokine (C-C motif) ligand 2	4.68	3.69
*Saa3*	serum amyloid A 3	4.67	6.25
*Cxcl10*	chemokine (C-X-C motif) ligand 10	4.57	4.24
*Ifi27l2a*	interferon, alpha-inducible protein 27 like 2A	4.44	1.73
*Ccl8*	chemokine (C-C motif) ligand 8	4.20	5.87
*Irf7*	interferon regulatory factor 7	4.16	1.80
*Sftpd*	surfactant associated protein D	3.84	13.99
*Saa2*	serum amyloid A 2	3.94	3.02
*Saa1*	serum amyloid A 1	3.79	5.72
*Oasl1*	2′-5′ oligoadenylate synthetase-like 1	3.69	2.96
*Il1f6*	interleukin 1 family, member 6	3.66	8.42
*Oas1a*	2′-5′ oligoadenylate synthetase 1A	3.60	1.86
*Ifi27*	interferon, alpha-inducible protein 27	3.53	1.93
*Ccl12*	chemokine (C-C motif) ligand 12	3.50	1.50
*Oas1f*	2′-5′ oligoadenylate synthetase 1F	3.34	2.05
*Ccl3*	chemokine (C-C motif) ligand 3	3.17	1.94
*Ifit2*	interferon-induced protein with tetratricopeptide repeats 2	3.05	2.52
*Tlr2*	Toll-like receptor 2	2.90	2.27
*Saa4*	serum amyloid A 4	2.89	3.12
*Tnfrsf1b*	tumor necrosis factor receptor superfamily, member 1b	2.86	1.88
*Il1rn*	interleukin 1 receptor antagonist	2.84	11.50
*B2m*	beta-2 microglobulin	2.76	2.04
*Ccl9*	chemokine (C-C motif) ligand 9	2.56	2.08
*Ifit1*	interferon-induced protein with tetratricopeptide repeats 1	2.54	2.33
*Gbp6*	guanylate binding protein 6	2.50	2.24
*Oas1d*	2′-5′ oligoadenylate synthetase 1D	2.45	4.78
*C4b*	complement component 4B	2.34	1.86
*Ltc4s*	leukotriene C4 synthase	2.31	2.76
*Ccl5*	chemokine (C-C motif) ligand 5	2.18	2.61
*Mpeg1*	macrophage expressed gene 1	1.57	1.57
*Cebpb*	CCAAT/enhancer binding protein (C/EBP), beta	1.35	1.44
*Adgre1*	adhesion G protein-coupled receptor E1	2.15	1.87
Fibrosis marker			
*Col1a1*	collagen, type I, alpha 1	2.31	1.69
*Col3a1*	collagen, type III, alpha 1	2.50	1.75
*Col17a1*	collagen, type XVII, alpha 1	2.51	3.55
*Col2a1*	collagen, type II, alpha 1	2.17	1.59
*Col12a1*	collagen, type XII, alpha 1	2.26	2.13
*Tnc*	tenascin C	2.08	2.06
*Areg*	Amphiregulin	2.01	8.97
*Itgav*	integrin alpha V	5.06	1.69
*Fn1*	fibronectin 1	3.31	2.89
*Timp1*	tissue inhibitor of metalloproteinase 1	4.66	3.01
*Mmp2*	matrix metallopeptidase 2	2.30	2.72
*Mmp3*	matrix metallopeptidase 3	5.07	3.46
*Fbn1*	fibrillin 1	2.41	2.53
*Atf3*	activating transcription factor 3	2.70	2.45
*Lox*	lysyl oxidase	2.33	2.05
Cellular senescence and apoptosis			
*Rprm*	reprimo, TP53 dependent G2 arrest mediator candidate	1.24	1.46
*Trp53inp1*	transformation related protein 53 inducible nuclear protein 1	2.16	3.87
*Tnfrsf10b*	tumor necrosis factor receptor superfamily, member 10b	1.61	4.64
*Cdkn1a*	cyclin-dependent kinase inhibitor 1A (P21)	12.22	15.15
*Ddias*	DNA damage-induced apoptosis suppressor	3.19	3.71
*Bcl2a1b*	B cell leukemia/lymphoma 2 related protein A1b	1.82	1.76
*Casp12*	caspase 12	1.43	1.75
*Aen*	apoptosis enhancing nuclease	1.43	1.66
*Casp4*	caspase 4	1.56	1.57
*Naip1*	NLR family, apoptosis inhibitory protein 1	4.49	2.60
*Bcl2a1c*	B cell leukemia/lymphoma 2 related protein A1c	2.05	1.65
*Bak1*	BCL2-antagonist/killer 1	1.49	1.50
*Bbc3*	BCL2 binding component 3	2.23	2.75
*Top2a*	topoisomerase (DNA) II alpha	11.37	3.92
*Bub1*	BUB1, mitotic checkpoint serine/threonine kinase	8.76	4.72
*Bub1b*	BUB1B, mitotic checkpoint serine/threonine kinase	4.76	1.86
*Chek1*	checkpoint kinase 1	1.98	3.36
*Mad2l1*	mitotic checkpoint component Mad2	1.95	1.97

**Table 2:** Primer sequences for qPCR.

Target Gene	Sequence (5′–3′)	
*L19*	Forward Reverse	GGCATAGGGAAGAGGAAGG GGATGTGCTCCATGAGGATGC
*Saa3*	Forward Reverse	AAGGGTCTAGAGACATGTGG ACTTCTGAACAGCCTCTCTG
*EMR1 (Adgre1)*	Forward Reverse	ATTGTGGAAGCATCCGAGAC GTAGGAATCCCGCAATGATG
*TNFα*	Forward Reverse	CGTCGTAGCAAACCACCAAG TTGAAGAGAACCTGGGAGTAGACA
*Mpeg1*	Forward Reverse	GCTTGCCTCTGCATTTCTTC TCTTCTGCTCCAGGTTTTGG
*CEBPβ*	Forward Reverse	GAAGACGGTGGACAAGCTGA TGCTCCACCTTCTTCTGCAG
*Ccl2 (MCP-1)*	Forward Reverse	GGTCCCTGTCATGCTTCTGG CCTTCTTGGGGTCAGCACAG
*Col1a1*	Forward Reverse	CCCAAGGAAAAGAAGCACGTC ACATTAGGCGCAGGAAGGTCA
*NOX2*	Forward Reverse	AGCTATGAGGTGGTGATGTTAGTGG TGCACAGCAAAGTGATTGGC
*TGFβ*	Forward Reverse	GGCACCATCCATGACATGAA TTCTCTGTGGAGCTGAAGCAAT

## Data Availability

Not applicable.

## Supplementary Materials

The following supporting information can be downloaded at: https://www.mdpi.com/article/10.3390/ijms23020899/s1.
