# Supplementary material for: Serum Amyloid A3 Promoter-Driven Luciferase Activity Enables Visualization of Diabetic Kidney Disease
Source: Int J Mol Sci. 2022 Jan 14;23(2):899. doi: 10.3390/ijms23020899 (PMC8779903; doi:10.3390/ijms23020899)
Supplement: Supplementary file 1 [file ijms-23-00899-s001.zip › ijms-1548837-supplementary.pdf]

Supplementary Materials

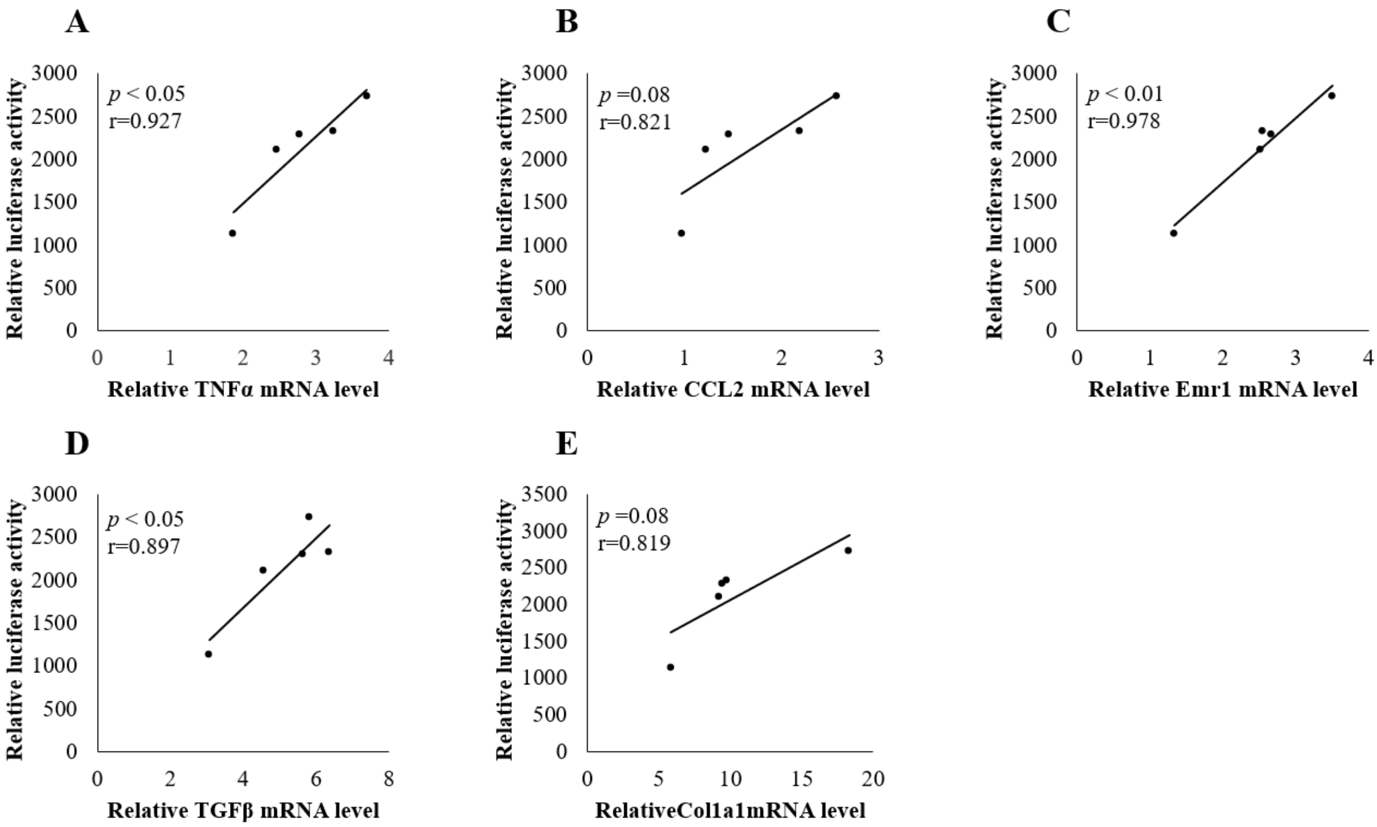

Figure S1. Positive correlation between luciferase activity and mRNA expression levels of TNFα, CCL2, Emr1, TGFβ, and Colla1 in two moderate-dose STZ-induced DN model (n = 5). The relative mRNA expression levels of TNFα, CCL2, Emr1, TGFβ, and Colla1 in kidney tissue were determined by quantitative PCR and normalized to L19 mRNA level (n = 5). Meanwhile the in vivo luciferase activities were determined by quantitative analysis of bioluminescence intensity from two moderate dose STZ-induced mouse kidneys (n = 5). Pearson's correlation coefficient showed a positive correlation between luciferase activity (promoter activity) and mRNA expression levels of TNFα (**A**), CCL2 (**B**), Emr1 (**C**), TGFβ (**D**), and Colla1(**E**) in kidney tissue.
